# Pulmonary Arterial Stiffness: An Early and Pervasive Driver of Pulmonary Arterial Hypertension

**DOI:** 10.3389/fmed.2018.00204

**Published:** 2018-07-18

**Authors:** Wei Sun, Stephen Y. Chan

**Affiliations:** Division of Cardiology, Department of Medicine, Center for Pulmonary Vascular Biology and Medicine, Pittsburgh Heart, Lung, Blood, and Vascular Medicine Institute, University of Pittsburgh School of Medicine and University of Pittsburgh Medical Center, Pittsburgh, PA, United States

**Keywords:** pulmonary arterial hypertension, arterial stiffness, endothelium, extracellular matrix, vascular metabolism

## Abstract

Pulmonary arterial hypertension (PAH) is a historically neglected and highly morbid vascular disease that leads to right heart failure and, in some cases, death. The molecular origins of this disease have been poorly defined, and as such, current pulmonary vasodilator therapies do not cure or reverse this disease. Although extracellular matrix (ECM) remodeling and pulmonary arterial stiffening have long been associated with end-stage PAH, recent studies have reported that such vascular stiffening can occur early in pathogenesis. Furthermore, there is emerging evidence that ECM stiffening may represent a key first step in pathogenic reprogramming and molecular crosstalk among endothelial, smooth muscle, and fibroblast cells in the remodeled pulmonary vessel. Such processes represent the convergence of activation of a number of specific mechanoactivated signaling pathways, microRNAs, and metabolic pathways in pulmonary vasculature. In this review, we summarize the contemporary understanding of vascular stiffening as a driver of PAH, its mechanisms, potential therapeutic targets and clinical perspectives. Of note, early intervention targeting arterial stiffness may break the vicious cycle of PAH progression, leading to outcome improvement which has not been demonstrated by current vasodilator therapy.

## Introduction

Pulmonary hypertension (PH) is defined as an elevation of the mean pulmonary artery pressure above 25 mmHg at rest. It can be divided into 5 clinical groups according to the World Health Organization (WHO) Classification of PH, last updated in 2013 (1) and scheduled to be revised later in 2018. Briefly, Group 1 PH is pulmonary arterial hypertension (PAH), often familial or heritable, related to connective tissue disease or toxins, with unknown underlying etiology (idiopathic PH). Group 2 refers to PH caused by left heart disease (pulmonary vein hypertension). Group 3 includes pulmonary hypertension resulting from lung diseases or hypoxia, such as chronic obstructive lung disease or sleep apnea. Group 4 disease is related to chronic thromboembolic obstruction in pulmonary arteries. Group 5 includes other less-common causes such as hematological or metabolic disorders that do not fit into any of the other four groups. The different groups of PH differ not only in fundamental pathogenesis but also patients' prognosis and corresponding treatment strategies. As a severe form of PH, PAH (WHO clinical Group 1) is a devastating condition characterized by progressive pulmonary vascular remodeling and gradual obstruction of pulmonary arterioles (at times, from pathognomonic plexiform lesions), resulting in increased pulmonary vascular resistance and pressure. The increase in pulmonary arterial pressure can lead to right ventricular heart hypertrophy, failure, and premature death.

PAH has idiopathic, heritable, and comorbid etiologies, associated with other chronic diseases. Although PAH is a highly morbid disease, it has been historically neglected since symptoms such as dyspnea and right heart failure often present late in disease. The onset of PH symptoms is unfortunately associated with a markedly impaired prognosis, and the historical survival rate of PAH without treatment is only 34% at 5 years (2). The current treatment options for PAH—including calcium-channel blockers, prostanoids, endothelin receptor antagonists, and phosphodiesterase inhibitors/soluble guanylyl cyclase agonists—primarily target pulmonary vascular dilation, thus lowering pulmonary arterial pressure, providing symptom relief and prolonging of the time to clinical deterioration. These treatments, however, are not curative and do not stop or reverse this disease. This may stem from the fact that these medications largely do not target to molecular origins of PAH (3), many of which still remain poorly defined. As such, there are obvious and critical needs, both scientifically and clinically, for advanced insights into the mechanisms of PAH pathogenesis in order to identify novel biomarkers for the early detection of PAH, to offer valid prognosis estimation, and to define novel targets for specific molecular therapeutics.

In this article, we will focus on the emerging appreciation of pulmonary vascular and extracellular matrix (ECM) stiffening as a distinct and early initiating cause of PAH. We will summarize the current understanding of pulmonary vascular stiffening, which serves not only as a consequence of PAH but also a critical driver of disease. We will review the progress in the studies on the mechanisms of pulmonary vascular stiffening, the potential therapeutic targets, and relevant clinical perspectives.

## Pulmonary arterial stiffening and ECM remodeling in PAH

### Pulmonary arterial remodeling and stiffening

Pulmonary arterial stiffening is a key component in the pathogenesis of PAH. Stiffness occurs in the proximal and distal pulmonary arteries in multiple subtypes of PAH (4, 5), and stiffness can serve as an index of disease progression (6). At the histologic level, pulmonary vessel stiffening in PAH is characterized by the activation and proliferation of all cellular subtypes in the three layers of the pulmonary arterial wall, including endothelial cells of the tunica intima, vascular smooth muscle cells of the tunica media, as well as adventitial fibroblasts. Spatially and functionally associated with the endothelial cell layer in the intima, the fibroblasts and myofibroblasts in the media and adventitia are activated, resulting in the synthesis and stromal deposition of ECM components. Within the tunica media, activated smooth muscle cells together with myofibroblasts derived from the adventitia can also migrate into the endothelial cell layer. As a result of this complex interplay between cells and ECM components, a concentric hyperplasia of the tunica intima develops (7). Of note, there is convincing evidence to suggest the active interaction between inflammatory cells especially macrophages with pulmonary arterial cells which plays important roles in the pathogenesis of PAH (8). Interestingly, the histopathological feature of this remodeling can resemble allograft vasculopathy as found after heart transplantation (9). The expression of some fetal variants of certain cell adhesion modulating proteins such as fibronectin or tenascin-C, as well as the impact of fibroblast to myofibroblast trans-differentiation and vascular smooth muscle cell activation have been extensively described in both settings of allograft vasculopathy and PAH. This remodeling process in the pulmonary vasculature results in a progressive decrease in arterial compliance and an increase in vascular stiffening.

Clinically, pulmonary arterial stiffening can be assessed by pulse wave velocity measurement via non-invasive ultrasound or magnetic resonance imaging, based on the pulsatile characteristics of the pulmonary arteries. Pulse wave velocity is the speed of flow waves propagate along the lumen, which depend on the stiffness and dimensions of the blood vessels. Concomitant measurement of pulmonary arterial stiffness indices could allow for more precise prediction for risk of developing severe PAH and mortality, especially in the early stage of the disease (10). For example, in children with congenital heart disease, increased pulmonary arterial stiffening predicts the progression into advanced PAH even in the patients with low pulmonary vascular resistance, suggesting intrinsic pulmonary arterial stiffness may serve as an independent index that may enhance predictions of disease progression and survival (11).

Pulmonary arterial compliance (PACa), as measured invasively or more recently via non-invasive means, is another pulmonary arterial stiffening index (6, 12). PACa refers the change in pulmonary arterial cross-sectional area or volume over change in pressure. Unlike pulmonary arterial resistance, which is calculated by the pressure difference across the pulmonary vasculature under a certain cardiac output, PACa reflects the pulmonary arterial stiffness more directly. PACa is increasingly appreciated as a parameter of prognostic relevance in patients with various subtypes and severities of PH (13, 14). In chronic thromboembolic PH (WHO Group 4), reduced PACa is a determinant of poor functional capacity and is a marker of poor prognosis in the patients. Reduced PACa may even serve as the most prominent hemodynamic feature found in early stages of PAH. Furthermore, in PH due to left heart disease (WHO Group 2), reduced PACa has been found to offer prognostic value, both in those with elevated and normal pulmonary vascular resistance (15).

### ECM stiffening

At the histologic level, ECM remodeling plays a central role in pulmonary arterial stiffening. The ECM network provides biophysical support for various cells in the vessel wall, thus maintaining the mechanical stability and elastic recoil of the arteries. Biochemical and biophysical signals induced by the ECM direct vascular cellular function, differentiation, migration and apoptosis, enable intracellular communication, playing a decisive role in vascular development, remodeling processes and maintenance of vascular homeostasis, as previously reviewed (16). The pulmonary arterial ECM is highly dynamic, consisting mostly of collagens, elastins, and laminins as well as other components such as fibronectin, tenascin C, and glucosaminoglycans. Dynamic balance of the ECM is dictated by bidirectional alterations of proteolytic enzymes such as a disintegrin and metalloproteases (ADAMs), matrix metalloproteinases (MMPs), and their endogenous inhibitors, tissue inhibitors of metalloproteinases (TIMPs). In PAH, buildup of collagen, neutrophil-elastase and MMPs, along with decrease of their modulating counterparts TIMPs causes an imbalance of ECM turnover and subsequently a pathogenic alteration of the structure and stiffness of the pulmonary vessel wall. A landmark of this remodeling process, endothelial-to-mesenchymal transition, is controlled by a complex upstream intracellular signaling network including TGF-β, Wnt, and Notch pathways (17). The restoration of ECM kinetics has been promoted as a strategy to prevent or ameliorate vascular remodeling processes but only recently have the molecular control points of this balance begun to be clarified.

ECM remodeling and stiffening can be triggered by various PAH pathogenic factors, such as vascular injury, pro-inflammation factors, abnormal growth factor expression, and/or hypoxia exposure. It has been shown that accumulation of ECM is a key pathologic change notable across the vascular wall in PH (18). Vascular-specific serine elastase activity in the ECM has been implicated in PAH (19). In parallel with elastase activity, MMPs exhibit an elevated activity, especially MMP-2/9, leading to accelerated turnover of ECM and play an important role in the initial remodeling of ECM in pulmonary vessels in both patients and animal models of PAH (20–22). Since the function of MMPs is tightly controlled by endogenous inhibitors TIMPs, the loss of balance between MMPs and TIMPs has been shown to induce ECM remodeling in patients with idiopathic PAH. As an example, Wang et al. found that vascular collagen buildup promotes hypoxia-induced pulmonary vascular remodeling in both small and large arteries (23). ECM remodeling could also delay the recovery of pulmonary hemodynamics and subsequently worsen right ventricular function (24). In regard of pulmonary vascular adventitial remodeling, the expression of type I collagen in cultured fibroblasts is upregulated by hypoxia (25). In rodent hypoxia-induced PH models, the expression of ECM proteins, especially the collagens that contribute to arterial stiffening, has been found to be elevated, even after hypoxic stress has been removed. This process is thought to be mediated by impaired degradation of type I collagen (26).

Approaches to target ECM remodeling in experimental animal models have shown efficacy in reducing PAH. Zhang et al. demonstrated that the extension of arterial stiffening is alleviated by inhibiting 15-lipoxygenase (15-LO) and consequent collagen accumulation in pulmonary arteries in hypoxia mouse models (25). Alternatively, in monocrotaline (MCT)-induced PAH in rats, the activation of AMP activated protein kinase (AMPK) by the AMPK agonist metformin inhibited ECM remodeling in pulmonary arteries, thus reducing the elevated right ventricle systolic pressure and right ventricle hypertrophy (27). The mechanisms underlying AMPK-induced suppression of ECM remodeling in pulmonary arteries were coupled to decreased MMP-2/9 activity and TIMP-1 expression. Importantly, however, the known pleiotropy of AMPK agonism in PAH likely extends far beyond the direct effects on pulmonary vascular stiffness (28, 29). Thus, while these results suggest the potential therapeutic value of AMPK activation in PAH, these studies did not fully prove the causative role of matrix stiffening *per se* in this disease. Phosphodiesterase type 5 enzyme (PDE-5) inhibitors are widely used in the clinical management of PH through the mechanism of NO mediated vasodilatation. As mentioned above, it is generally thought that the vasodilators present no significant effect in stopping or reversing the progression of PAH. However, data exist that show the PDE-5 inhibitor sildenafil restores BMP signaling in cultured BMPR2 deficient smooth muscle cells, and also reduces pulmonary vascular remodeling in the MCT-induced PAH rat model (30).

## Arterial and ECM stiffening as early driver of PAH

Although the connection of pulmonary vascular stiffening/ECM remodeling to PAH has been well established, until recently, the question remained unanswered as to whether ECM remodeling represented simply an end-stage consequence of PAH. Contemporary studies, including work from our group, have advanced the concept that vascular stiffening and ECM remodeling are early and potent pathogenic triggers in PAH, occurring at time points prior to typical hemodynamic and histologic evidence of PAH or medial thickening (31–33). Several major mechanisms have been identified in this process, especially the activation of mechanosensitive metabolic pathways, the pro-inflammatory phenotype switch of activated fibroblasts and macrophages, and the involvement of certain groups of mechanosensitive microRNAs.

### Metabolic mechanisms involved in pulmonary vascular stiffening in PAH

Metabolic reprogramming in both the diseased pulmonary vasculature and right ventricle is increasingly appreciated as a crucial mediator of the pro-proliferative condition in PAH. Not only hypoxia but also other PAH-inducing factors, such as genetic mutations, congenital heart disease, scleroderma, and HIV infection, are all related to profound metabolic dysregulation in the pulmonary vasculature. For instance, broad metabolic reprogramming, beyond aerobic glycolysis and independent of hypoxia, has been identified through metabolomic screening in pulmonary endothelial cells carrying a bone morphogenetic protein receptor type 2 (BMPR2) mutation, a known genetic driver of PAH (34). The utilization and metabolism of glutamine have also been found to be increased in the course of rat right ventricle remodeling and human PAH (35).

The shift of energy production from mitochondrial oxidative phosphorylation to glycolysis chronically (Warburg-like effect), represents a central feature of the extensive reprogramming found in pulmonary arterial endothelial cells and smooth muscle cells in PAH, as reviewed previously (36). Furthermore, such metabolic reprogramming has also been linked to a pathophenotypic switch of both pulmonary vascular macrophages and fibroblasts which then can contribute extensively to pulmonary arterial stiffening and matrix remodeling in PAH. In both experimental hypoxic PH in rodents and human PAH, a model of cellular crosstalk has emerged whereby adventitial fibroblasts can recruit, retain, and activate naive macrophages into a hyper-proliferative, apoptosis-resistant, and proinflammatory phenotype (37–39). To drive this process in adventitial fibroblasts, pathogenic suppression of mitochondrial bioenergetics was found to be accompanied by increased mitochondrial fragmentation, resulting in less-efficient ATP synthesis, hyperpolarized mitochondria, and increased mitochondrial superoxide production. In contexts both dependent and independent of hypoxia, these changes resulted in a pro-oxidative and pro-inflammatory status in the pulmonary vascular micro-environment (38, 40), thus driving matrix alterations and resulting in pulmonary vascular stiffening. Metabolic reprogramming in activated macrophages has also been described in PAH, highly dependent on increased aerobic glycolysis, altered TCA cycle activity, and reduced mitochondrial respiration (Warburg-like effect). Such reprogramming has been linked to a number of distinct and complicated mechanisms involving a cohort of metabolic enzymes and accumulated metabolites, as well as transcriptional regulatory factors such as the hypoxia inducible factor (HIF)-1α and downstream signaling molecules such as STAT3 [see reviews by (41, 42)].

Of note, the energy production from the increased glycolysis alone is not sufficient to fulfill the metabolic demands for the cells that are actively proliferating during the process of vascular remodeling in PAH. The energy generated from the classic tricarboxylic acid (TCA) cycle is necessary to maintain the cellular reprogramming process. Additionally, large amounts of various precursor substrates are produced via TCA cycle activity. These substrates are critical for amino acid, carbohydrate and lipid biosynthesis which supports rapid cell growth and proliferation. In doing so, a large quantity of carbon intermediates are consumed continuously and requires replenishment. This replenishment of TCA carbon intermediates, also termed anaplerosis, depends on two major mechanisms. One is glutaminolysis, which deamidates glutamine via the enzyme glutaminase 1 (GLS1). The other pathway is the carboxylation of pyruvate to oxaloacetate, which is mediated by ATP-dependent pyruvate carboxylase. Particularly, GLS1-mediated glutaminolysis in the proliferating cells during pulmonary vascular remodeling serves as a critical mechanism to support not only the mobilization of cellular energy, carbon and nitrogen to maintain cellular biomass but also the metabolic switch from oxidative phosphorylation to glycolysis.

**Figure 1:**
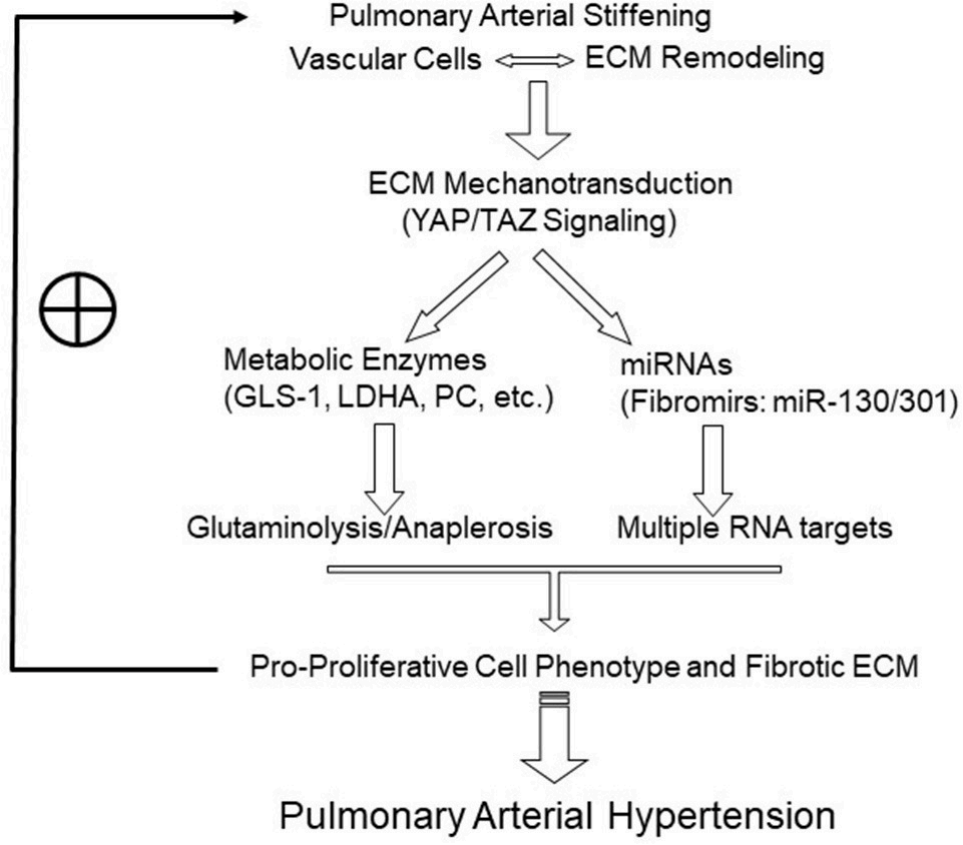
Model of the positive feedback loop and vicious cycle triggered by pulmonary arterial and matrix stiffening in PAH inception and development. Pulmonary arterial stiffening and matrix remodeling activate mechanosensitive signaling. A prominent example includes YAP/TAZ-dependent reprogramming, which induces the key metabolic enzymes to promote glutaminolysis and glycolysis and sustains the metabolic needs of hyperproliferative vascular cells. In the other hand, mechanoactive YAP/TAZ signaling also induces miRNAs that are related to tissue fibrosis and remodeling (“fibromirs”), through interacting with multiple, and potentially synergistic, target mRNAs. These mechanisms drive pro-proliferative vascular cell phenotypes and ECM remodeling and in turn further enhance mechanotransduction, forming a vicious cycle underlying the progression of PAH. ECM, extracellular matrix. The symbol ⊕ indicates a positive feedback effect to maintain the vicious cycle.

Recently, emerging evidence, including findings from our group, has demonstrated a link between matrix mechanotransduction (i.e., mechanisms by which cells sense and react to external mechanical forces and convert extracellular mechanical cues into intracellular signaling) and pulmonary vascular metabolic reprogramming, thus triggering the initiation and development of PAH (31, 33). We previously defined the pulmonary vascular function of YAP (Yes Associated Protein 1) and TAZ (Transcriptional Coactivator with PDZ-Binding Motif, or WWRT1), which serve as transcriptional coactivators in the Hippo signaling pathway. YAP/TAZ are mechanoactivated by stiff ECM in pulmonary vascular cell types and function as central regulators of cellular proliferation. They do so by activating the enzyme GLS1 which promotes glutaminolysis and anaplerosis and downstream effects on cellular proliferation, migration, and apoptosis among multiple vascular cell types in a timed and stage-specific manner. As such, YAP/TAZ-dependent control of glutaminolysis may act as a central mechanism of the pulmonary vascular dysfunction induced by the change of the extracellular environment in PAH (Figure 1) (33).

Besides YAP/TAZ–GLS1 activation, some additional mechanisms may also be involved in the metabolic switch in the setting of pulmonary arterial stiffening. The enzymes lactate dehydrogenase A (LDHA) and the ATP-dependent pyruvate carboxylase (PC) have both been identified as associated checkpoints in stiffness-induced alterations of glycolysis and anaplerosis, respectively (33). Furthermore, YAP/TAZ signaling has been linked to AMPK activation, resulting in the induction of aerobic glycolysis (43). These findings suggest broader control over metabolic reprogramming in PAH by YAP/TAZ and vascular stiffness. Future work will be important to delineate more completely the connections of pulmonary vascular stiffness and glutaminolysis with cellular reprogramming, crosstalk, and also the timed evolution of cellular function and identity in PAH. These findings may also endorse the application of novel pharmacologic agents targeting the metabolic effects of vascular stiffness to prevent or even reverse PAH, which will be further discussed below.

### MicroRNA-related mechanisms in pulmonary arterial stiffening

MicroRNAs (miRNAs) are non-coding RNAs that vary in length between 19 and 25 nucleotides. They regulate gene expression by affecting mRNA stability and translation into protein, and they serve as essential mediators of multiple cellular processes involving cell-cell and cell-matrix interactions (44). Although many crucial roles of miRNAs in the cardiovascular system have been well established, studies of miRNAs in PAH are still advancing (3, 45). Until recently, the interaction between miRNAs and pulmonary arterial stiffening/ECM remodeling had not been well established, and the targets and downstream mechanisms of miRNAs were largely unexplored.

Numerous miRNAs have been found to function in the pathogenesis and progression of PAH, as previously reviewed (46). Among them, the miR-130/301 family has been shown to regulate the systems-wide, proliferative and vasoconstrictive actions in pulmonary vasculature. The involvement of miRNAs in pulmonary ECM biology, their targets and mechanisms, have been recently studied as an early driver of PAH progression. Specifically, mechanosensitive YAP/TAZ signaling has been found to activate miR-130/301 to form a feedback loop, thus promoting PAH via ECM remodeling and vascular stiffening (31). The downstream mediators of the YAP/TAZ-miR-130/301 circuit relevant to ECM remodeling were found to involve predominantly the pro-proliferative PPARγ-APOE-LRP8-LOX pathway. Overexpression or knockdown of PPARγ or its direct target ApoE, disrupted miR130/301 mediated vascular collagen disposition and remodeling (31). Other secreted factors, such as fibroblast growth factor 2 (FGF2), interleukin-6 (IL-6), and endothelin 1 (EDN1), as well as miRNAs such as miR-21 and miR-27a, have further defined an overlapping molecular hierarchy important to the remodeled ECM in PAH [as reviewed in Negi and Chan (3)]. The early development of ECM remodeling in PAH and the positive feedback loop connecting the ECM to the YAP/TAZ-miR-130/301 circuit suggest the pathogenic relevance of this axis both early and late in disease as well as present a model of self-sustained propagation of ECM remodeling throughout the pulmonary vascular tree as PAH progresses.

Much future work remains in studying the roles of miRNAs in pulmonary arterial stiffening. It is likely that an even more complex and broad interactome is active among YAP/TAZ, a cohort of fibrosis-related miRNAs including miR-130/301, and their targets. For example, independent of YAP/TAZ, pulmonary vessel stiffening has also been found to be regulated by a miRNA-dependent process involving the Runt-related transcription factor 2 (Runx2) to promote vascular calcification (47). Moreover, mechanoactivation of YAP/TAZ independent of miR-130/301 can promote increased matrix deposition and stiffening, thus propagating vessel stiffness throughout the pulmonary vascular tree in PAH (48). Recent studies have implicated the control of metabolic, inflammatory, and proliferative regulatory programs in diseased adventitial fibroblasts in PAH to miR-124 (49, 50) and transcriptional regulators, such as the C-terminal binding protein-1 (CtBP1) (51) and the polypyrimidine tract binding protein 1 (PTBP1)-pyruvate kinase muscle (PKM) axis (49).

Thus, a set of mechanosensitive and ECM-related miRNAs (“fibromirs”) may comprise an important link in the complex regulation of ECM stiffening in PAH (Figure 1). Future work will be required to define additional mechanisms of RNA-dependent control of this process in PAH, specifically in regard to other aspects of ECM remodeling beyond biogenesis, such as matrix degradation and turnover.

## Novel translational approaches targeting pulmonary arterial stiffening in PAH

### Diagnostic and therapeutic targets in the ECM for PAH

Given the pathogenic importance of vascular ECM reorganization in PAH, investment is ongoing to identify matrix components that may serve either as biomarkers for diagnosis and prognostic estimation in PAH or as true therapeutic targets. The translational value of measuring fetal ECM components, such as tenascin-C variants, as biomarkers, both in tissue and in circulating blood, has been proposed (52, 53). Due to the stable extracellular deposition, these matrix components could also be considered as target molecules for specific therapeutic modulation in PAH. For example, fibronectin and tenascin-C have been considered as feasible molecular targets for antibody-based delivery of diagnostic agents (e.g., radionuclides) or direct therapeutics (i.e., bioactive payloads such as cytokines or small molecule inhibitors). Such agents, in particular immunocytokines or antibody-drug conjugates, have been successfully administered in a variety of animal models of neoplastic and non-neoplastic chronic-inflammatory diseases (54–56). Applications of these approaches in PAH could advance considerably in the near future.

### Therapeutic strategy targeting miRNA-dependent or metabolic mechanisms of pulmonary arterial stiffening

Multiple novel therapeutic gene targets in adventitial fibroblasts have shown promise recently in experimental rodent models of PH, including the CtBP1 inhibitor 4-methylthio-2-oxobutyric acid (MTOB) (51) and the PKM2 inhibitors TEPP-46 and shikonin (49) among others. Importantly, however, similar to targeting AMPK activity, the therapeutic effects of these pleiotropic agents may not solely be dependent on their effects on pulmonary vascular stiffness alone. On the other hand, the description of a true mechanosensitive YAP/TAZ-GLS1 circuit regulating glutaminolysis and cellular proliferation sets the stage for developing novel clinical management strategies in PAH that target pulmonary arterial stiffening more directly. As a historical example, the lysyl oxidase (Lox) inhibitor β-aminopropionitrile (BAPN) has been used to improve both hemodynamic and histologic indices of PAH in experimental rodent models, thus reinforcing the therapeutic feasibility of targeting collagen cross-linking and ECM reprogramming in PH (33, 57–59). Consistent with more recent evidence supporting the important roles of YAP and GLS1 in the pathogenesis of PAH, a YAP inhibitor such as verteporfin (33, 60), an oligonucleotide inhibitor of the miR-130/301 family (61), as well as GLS1 inhibitors such as CB-839 and C968 (33) have all been shown to mediate robust improvement of rodent PAH. Downstream of YAP, a liver X receptor (LXR) agonist GW3965, which upregulates ApoE level when administered orally to hypoxia treated mice, was shown to ameliorate ECM remodeling and PAH through interaction with YAP/TAZ-miR-130/301 circuit *in vivo* (31, 62). Importantly, verteporfin is already approved for use intravenously in treatment of age-related macular degeneration (63). Cyclic YAP-like peptides that interrupt YAP-TEAD interactions in oncogenesis (64) could also be applied to PAH. Moreover, CB-839, acting as oral GLS1 inhibitor, is developed for cancer therapy and currently under evaluation in an early human clinical trial (Clinical Trial NCT02071862) (65). Notably, since LXR along with the sterol regulatory element-binding protein (SREBP) also act together to mediate the actions of AMPK (66), there may exist an even greater interdependence and convergence of YAP with AMPK than already appreciated. Therefore, a strategy of repurposing these inhibitors for YAP, GLS1, or miR-130/301, potentially along with a LXR agonist and/or AMPK modulator, may provide a rare opportunity to offer novel “matrix” therapeutics for PAH without the delay of needing to develop new inhibitors *de novo*.

## Summary

ECM remodeling and pulmonary arterial stiffening have long been associated with PAH. However, only recently have we begun to appreciate that pulmonary vascular and ECM stiffening act as crucial early triggers in pathogenesis of this mysterious disease. Emerging evidence has demonstrated that pulmonary vascular stiffening activates mechanosensitive signaling such as YAP/TAZ signaling which in turn regulates downstream miRNAs and metabolic targets essential for PAH development (Figure 1). Further understanding of this and other novel mechanisms related to pulmonary arterial stiffening will strengthen the development of diagnostic, therapeutic, and potentially preventative, approaches targeting the early initiation of arterial stiffness. As such, it is hoped that the vicious cycle of PAH progression may be broken or entirely avoided, thus leading to disease prevention or reversal, which has not been possible thus far with current therapies.

## Author contributions

All authors listed have made a substantial, direct and intellectual contribution to the work, and approved it for publication.

### Conflict of interest statement

SYC has served as a consultant for Actelion (Significant), Gilead, Pfizer, and Vivus (Modest). The remaining author declares that the research was conducted in the absence of any commercial or financial relationships that could be construed as a potential conflict of interest.
